# A Single Dose of Beetroot Gel Rich in Nitrate Does Not Improve Performance but Lowers Blood Glucose in Physically Active Individuals

**DOI:** 10.1155/2017/7853034

**Published:** 2017-01-24

**Authors:** Julia Vasconcellos, Diego Henrique Silvestre, Diego dos Santos Baião, João Pedro Werneck-de-Castro, Thiago Silveira Alvares, Vânia M. Flosi Paschoalin

**Affiliations:** ^1^Instituto de Química, Universidade Federal do Rio de Janeiro, 21941-909 Rio de Janeiro, RJ, Brazil; ^2^Instituto de Biofísica Carlos Chagas Filho e Escola de Educação Física e Esporte, Universidade Federal do Rio de Janeiro, 21941-902 Rio de Janeiro, RJ, Brazil; ^3^Division of Endocrinology and Metabolism, Rush University Medical Center, Chicago, IL 60612, USA; ^4^Instituto de Nutrição, Núcleo Básico de Nutrição e Dietética, Universidade Federal do Rio de Janeiro, 27979-000 Macaé, RJ, Brazil

## Abstract

*Background*. Beetroot consumption has been proposed to improve exercise performance, since the nitrate content of this food is able to stimulate the synthesis of nitric oxide.* Objective*. The acute effect of 100 g of a beetroot gel containing ~10 mmol of nitrate was tested on the nitric oxide synthesis, on metabolic and biochemical parameters, and on performance in physically active individuals.* Methods*. Through a double blind, crossover, placebo-controlled study, 25 healthy runners ingested a single dose of beetroot and placebo gels. Participants performed an aerobic exercise protocol on a treadmill (3 min warm-up of 40% peak oxygen consumption, 4 min at 90% of gas exchange threshold I and 70% (Δ) maximal end speed until volitional fatigue).* Results*. Urinary levels of nitrite and nitrate increased after 90 min of beetroot gel ingestion. Plasma glucose concentrations lowered after the exercise and the decrease was maintained for 20 min. Systolic and diastolic blood pressures, serum cortisol, and blood lactate were not altered after the beetroot gel ingestion compared to a placebo gel.* Conclusion*. The single dose of beetroot gel provoked an increase of nitric oxide synthesis although no improvement on the physical performance of athletes during aerobic submaximal exercise was observed.

## 1. Introduction

The interest of the athletic population in sports nutrition as a way to boost physical performance has increased exponentially [1, 2]. Several studies have been conducted on the influence of selective nutrition in improving the health and physical performance of individuals [3, 4].

One nutrient that is gaining prominence is dietary nitrate (NO_3_^−^). Endogenous NO_3_^−^ synthesis occurs through the L-arginine/nitric oxide (NO) pathway. The nitric oxide synthase (NOS) enzyme alongside its cofactors catalyzes the formation of L-citrulline and NO from L-arginine. Once synthesized, NO is rapidly oxidized to nitrite (NO_2_^−^) or by ceruloplasmin and the NO_2_^−^ formed also can be oxidized or suffer action of oxyhemoglobin to generate NO_3_^−^. Furthermore, NO can react directly with oxyhemoglobin to generate NO_3_^−^ [5–7]. Moreover, the major exogenous source for the acquisition of NO_3_^−^ is via the dietary route. In a Western diet, most of dietary NO_3_^−^ intake is obtained from vegetables [8].

Beetroot (*Beta Vulgaris L*. species) is a vegetable rich in nutrients, such as sugars, phenolics compounds, and ascorbic acid, and considered as a dietary NO_3_^−^ source [9, 10]. The NO_3_^−^ ingested through the diet can be reduced to nitrite (NO_2_^−^) in the oral cavity by anaerobic commensal bacteria expressing the NO_3_^−^ reductase enzyme, and the NO_2_^−^ formed can be converted to NO in blood and tissues through enzymatic and nonenzymatic pathways [11]. By stimulating NO synthesis, the NO_3_^−^ from beetroot has been proposed as capable of restoring endothelial function [6, 12] and enhancing the blood perfusion [13] and performance in elite athletes [14–16]. For this reason, the dietary NO_3_^−^ ingestion from beetroot, before athletic or sporting competitions, has become increasingly popular since it could be a legal and healthy way to enhance performance in elite athletes.

Several studies point to improvements in physical performance during endurance exercises, such as running and cycling, after the ingestion of NO_3_^−^ in doses ranging from 5 to 16.8 mmol [14, 17, 18]. A significant increase in NO synthesis (evaluated by plasma NO_3_^−^ and NO_2_^−^) associated with an increase in muscle contractile efficiency and reduction in O_2_ cost was observed. Additionally, an increased tolerable duration of moderate and severe-intensity exercises was also noticed. Taken together, these results indicate that dietary NO_3_^−^ is capable of increasing the efficiency of the oxidative metabolic pathways to provide energy substrates for muscle contraction during exercise. However, the most positive results in physical performance were observed when the dietary NO_3_^−^ supplementation from beetroot starts at approximately 6 days before the intervention [15, 19–21], while no effects were observed after a single dose of NO_3_^−^ supplementation [22]. There is still no consensus on the single dose effect from NO_3_^−^ enriched-beetroot formula, except for juice administration in recreational athletes subjected to endurance test on a treadmill in sea level.

Dietary NO_3_^−^ supplementation from beetroot is challenging with regard to the form of the beetroot administration. Foods in the gel form have certain technological advantages, such as being easy to store at room temperature, to carry and ingest, and to tolerate by athletes. Furthermore, gel foods tend to have higher concentrations of nutrients in a reduced volume, which favors greater access of the dietary nutrients to the cells [9]. In this context, the present study aims to analyze the biochemical and metabolic changes and effects on the performance of physically active individuals resulting from the ingestion of a single dose of nutritional beetroot gel rich in dietary NO_3_^−^, to evaluate the effects of this nutritional strategy on the practice of endurance sports.

## 2. Methods

### 2.1. Participants

**Figure 1:**
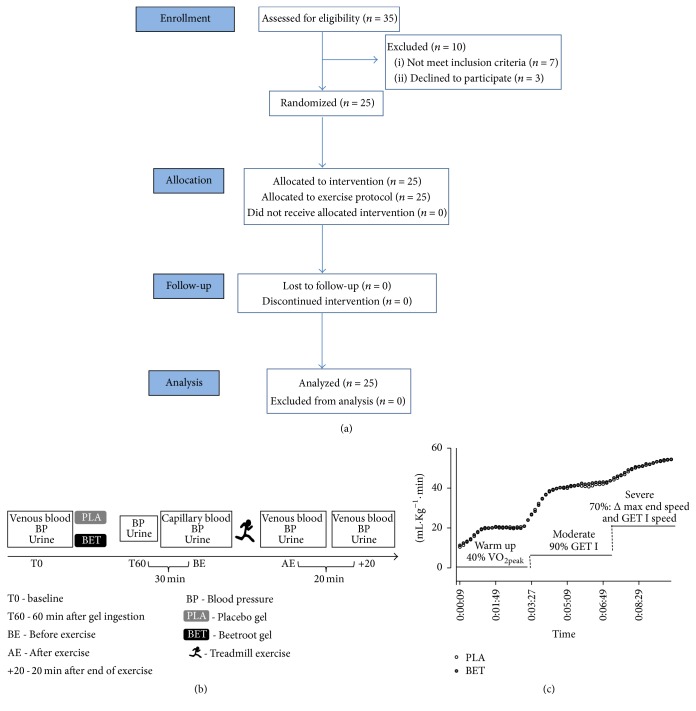
Summary of the treadmill exercise protocol. (a) CONSORT flow diagram, (b) acute aerobic exercise protocol, and (c) VO_2_ collections performed on the 3rd and 4th visits, of the BET and PLA treatment volunteers.

Twenty-five healthy runners (11 women: 36.27 ± 5.57 yr, 58.19 ± 3.54 kg, 26.13 ± 4.38% BF, 52.79 ± 4.57 mL·kg^−1^·min^−1^  VO_2peak_ and 14 men: 35.36 ± 6.59 yr, 73.50 ± 8.27 kg, 16.24 ± 2.91% BF, 64.31 ± 4.71 mL·kg^−1^·min^−1^  VO_2peak_) were enrolled in the study and classified as having an excellent VO_2peak_ according to the American College of Sports Medicine (99% percentile). The inclusion criteria were to be physically active and have more than one year of running experience, supervised by a physical educator. The exclusion criteria were any known cardiovascular, pulmonary, renal, and metabolic diseases, musculoskeletal injuries, use of steroids or nutritional supplements, smokers, intolerance or allergies to any of the beetroot, and/or the natural red dye components (Figure 1(a)). All participants were fully informed of the nature of the investigation and informed consent in accordance with Resolution number 446/12 of the National Health Council. All experimental procedures were performed in accordance with the ethical standards of Helsinki and approved by the institutional ethics committee (Hospital Universitário Clementino Fraga Filho, Rio de Janeiro, under number 21826513.3.0000.5257).

On the day before the visits, the participants were instructed to avoid the use of antibacterial mouthwash, intake of alcohol, caffeine, and foods rich in NO_3_^−^ and NO_2_^−^ for 24 h.

### 2.2. Study Design

Participants performed 4 visits with seven days washout between the last two visits. During the 1st visit, participants were familiarized with the aerobic treadmill protocol and advised to fast for 8 h and follow their ordinary exercise training but avoiding intense exercise 24 h prior the 2nd, 3rd, and 4th visits. On the 2nd visit, the VO_2peak_ of each participant was evaluated using an indirect calorimeter model CPX (VacuMed®, CA, EUA) [23]. On the 3rd and 4th visits (Figure 1(b)), after overnight fasting, venous blood, urine samples and blood pressure values for the baseline measurements were collected. Subsequently, each participant received two single gels in black sachets with 50 g each of either beetroot-based nutritional (BET) or a placebo gel (PLA) and 300 mL of tap water. Subjects consumed 100 g of either BET or a PLA gel in a double blind, crossover, placebo-controlled and randomized experimental design within 15 min. No adverse effects were reported.

### 2.3. Exercise Protocol

This aerobic exercise protocol performed on 3rd and 4th visits and VO_2_ collections performed on the volunteers of the BET and PLA treatments was carried out as previously described [14] but adjusted to treadmill (Figure 1(c)). Ventilation (VE), inspired oxygen, and expired CO_2_ were measured during the treadmill run, and measurements were averaged over 10s intervals. Also, the respiratory quotient (RQ) was measured. The* gas exchange threshold I *(GET I) was determined using the V-slope method [24] as the first disproportionate increase in CO_2_ production (VCO_2_) relative to the increase in VO_2_ and subsequently verified by an increase in the ventilatory equivalent for VO_2_ (VE/VO_2_), with no increase in VE/VCO_2_. The test began with a 3 min warm-up at 40% of VO_2peak_ followed by 4 min of moderate intensity at 90% of the GET I and a subsequent severe intensity to volitional fatigue at 70% of the difference (Δ) between GET I and VO_2peak_, using the following equation:

(1) Final  speed=maximum  final  speed  in  VO2peak−GET  I×70%+GET  I.

The rating of perceived exertion (RPE) measurements was performed by using an adapted Borg 0 to 10 point scale [25]. RPE were recorded at every minute after completing the warm-up stage.

### 2.4. Systolic and Diastolic BP and Heart Rate (HR)

Three measurements of supine blood pressure of the brachial artery were taken using an automated sphygmomanometer (Cardiomed®, Curitiba, BR) applied to the right arm after 5 min rest. Exercise HR was measured during the test by a cardio tachometer, model T31 (Polar®, Kempele, FIN). HR was recorded at every minute after completing the warm-up stage.

### 2.5. Blood and Urine Samples

Blood drawn from the antecubital vein was collected in tubes containing EDTA and a gel clot activator and centrifuged (at 1,500 ×g for 10 min at 4°C) to separate plasma and serum. The plasma was stored at −80°C for subsequent analyses. Capillary blood was obtained by a transcutaneous puncture. Urine samples were taken in appropriate sterile containers, aliquoted, and stored at −80°C for subsequent analyses.

### 2.6. Urinary NO_2_^−^ and NO_3_^−^ Analyses (NO Synthesis)

Urinary NO_2_^−^ and NO_3_^−^ levels were determined as previously described [11] using a high-performance liquid chromatography (HPLC) system. Urine samples were diluted at a ratio of 1 : 100 for NO_2_^−^ and 1 : 2000 for NO_3_^−^ analysis in distillated and deionized water. Then, the samples were filtered through 10 kDa cutoff ultrafilters (Vivaspin 500; GE® Healthcare Life Sciences; Uppsala, Sweden) at 14.000 ×g for 15 min to remove high molecular weight proteins. For the NO_2_^−^ analysis, 100 *μ*L of filtered sample was incubated at 24°C with 10 *μ*L of 316 mmol·L^−1^ of 2.3-diaminonaphthalene (DAN) in 0.62 mol·L^−1^ HCl for 10 min followed by the addition of 5 *μ*L of 2.8 mol·L^−1^ NaOH and immediately analyzed by HPLC. For the NO_3_^−^ analysis, NO_3_^−^ sample was enzymatically converted to NO_2_^−^ through the addition of 10 *μ*L of nitrate reductase (EC 1.6.6.2* Aspergillus* species, Roche Diagnostics, Mannheim, Germany) and 10 *μ*L of 120 mM NADPH (Roche Diagnostics, Mannheim, Germany). Then, the sample was incubated at room temperature for 1 h. After conversion of NO_3_^−^ to NO_2_^−^, this solution is used directly for the analysis of NO_2_^−^ using HPLC system. The HPLC device was equipped with a 5 *μ*m reversed-phase C8 column (150 × 4.6 mm, ID), a 5 *μ*m reversed-phase C18 guard column (50 × 4.6 mm, ID) (Ascentis®, Bellefonte, PA, USA), and a fluorescence detector model RF-10AXL (Shimadzu®, Kyoto, JPN). The mobile phase (1.3 mL·min^−1^) was 15 mmol·L^−1^ sodium phosphate buffer (pH 7.5) and methanol (50 : 50, v/v) at a gradient elution.

### 2.7. Colorimetric Analyses

Urea and creatinine levels in urine and serum cortisol were determined by colorimetric methods in 96-well plates (absorbance at 570 nm) on a VICTOR™ X4 spectrophotometer (PerkinElmer®, MA, USA), using commercially available kits, as follows: Urea Assay Kit MAK006, Creatinine Assay Kit MAK080, and Cortisol ELISA SE120037, purchased from Sigma-Aldrich® Chemical Co, MO, USA.

Capillary blood glucose and lactate were analyzed within 30 s after collection on a Yellow Springs Instrument (YSI Life Sciences Inc, OH, USA).

### 2.8. Beetroot-Based Nutritional (BET) and PLA Gels

Beetroots belonging to the Chenopodiaceae family and* Beta vulgaris L*. species were purchased at a local market in Rio de Janeiro city (Southeastern Brazil - 22°54′S 43°10′W). The gels formulation was produced as previously described [9]. Beetroots were washed in tap water, sanitized with a chlorine solution, and put into a centrifuge blender (model CE700, Black & Decker®). The juice produced has 91.55 ± 0.75% of moisture and it was stored at −20°C for further use. Also, other beetroots were washed in tap water, sanitized with a chlorine solution, sliced (3–8 cm wide and 2–4 mm thick), frozen at −20°C for 48 h, and freeze dried in a Liotop P1040 model (Liobras, São Paulo, Brazil). Then, these beetroots were crushed in a portable blender (Blender Pratic® cadence) to produce beetroot powder. To produce 100 g of BET, 90 mL of beetroot juice was mixed with 16.8 g of beetroot powder, 2.8 g of carboxymethylcellulose (C4888 Sigma-Aldrich Co, MO, USA), and 1 mL of artificial orange flavor.

The PLA was prepared by depleting the NO_3_^−^ from the beetroot juice in a NO_3_^−^ specific A-520E anion-exchange resin (Purolite®, PA, USA). After 1 h on an orbital shaker, the juice suspension was loaded onto a sterile chromatography glass column (3 cm × 55 cm, ID) and eluted with a vacuum pump. Fuji apple (*Malus pumila *species) puree was prepared by liquefying the apples in a portable blender (Cadence Co, RS, BR). To produce 100 g of PLA gel, 90 mL of NO_3_^−^-depleted beetroot juice, 16.8 g of apple puree (in substitution of beet chips), 2.8 g of carboxymethylcellulose, and 1 mL of artificial orange flavor were mixed.

### 2.9. Dietary NO_3_^−^ and NO_2_^−^ Contents of BET and PLA Gels

Analyses of NO_3_^−^ and NO_2_^−^ contents of gels were performed by weighing 1 g of gel samples and then dissolving in 8 mL of water. Samples were filtered through a 0.45 *μ*m cellulose membrane filter (MF Millipore®, Darmstadt, GER); NO_3_^−^ and NO_2_^−^ analyses were performed as previously described [8] using HPLC. All samples were analyzed in triplicate (4 × 3) and the results were expressed in mmol of NO_2_^−^  · 100 g^−1^ and NO_3_^−^  · 100 g^−1^.

### 2.10. Statistical Analyses

All assumptions for using parametric tests were verified. The normality of all data was tested by the Shapiro-Wilk test. One-way (ANOVA) analysis of variance was performed with repeated measures to identify differences in the dietary NO_2_^−^ and NO_3_^−^ contents between BET and PLA gels. Analyses of cortisol, HR, and RQ between PLA and BET treatments were assessed using two-factor repeated measures ANOVA 2 × 3 for condition × time, of data collection (0, AE, +20 min). Analysis of Borg scale was conducted using one-factor repeated measures ANOVA to establish differences between conditions. Differences in urinary NO_2_^−^ and NO_3_^−^, urea, creatinine, and BP (systolic and diastolic) between PLA and BET treatments were assessed using two-factor repeated measures ANOVA 2 × 5 for condition × time, of data collection (0, 60, 90, BE, AE, +20, min). Analyses of lactate and blood glucose, between PLA and BET treatments, were assessed using two-factor repeated measures ANOVA 2 × 4 for condition × time, of data collection (0, 90, BE, AE, +20 min). Post hoc analysis of significant within-subject effects was performed with adjustments for multiple comparisons using the Bonferroni correction. Differences in exercise time and VO_2_ were analyzed using two-tailed, paired samples *t*-tests. The null hypothesis was rejected when *p* < 0.05. Statistical procedures were completed using Graphpad Prisma for windows version 5.

## 3. Results

### 3.1. Gel NO_2_^−^ and NO_3_^−^ Contents

The NO_3_^−^ content of PLA and BET were 0.33 ± 0.15 and 9.92 ± 1.97 mmol·100 g^−1^ (*p* = 0.014), respectively. NO_2_^−^ content was insignificant (<10 *μ*mol·100 g^−1^) in both BET and PLA gels. The gels were well tolerated and only beeturia (red urine) was reported. This condition does not represent any health problem and is expected in studies of this nature. After the study period, the volunteers did not report beeturia anymore.

### 3.2. NO Synthesis

**Figure 2:**
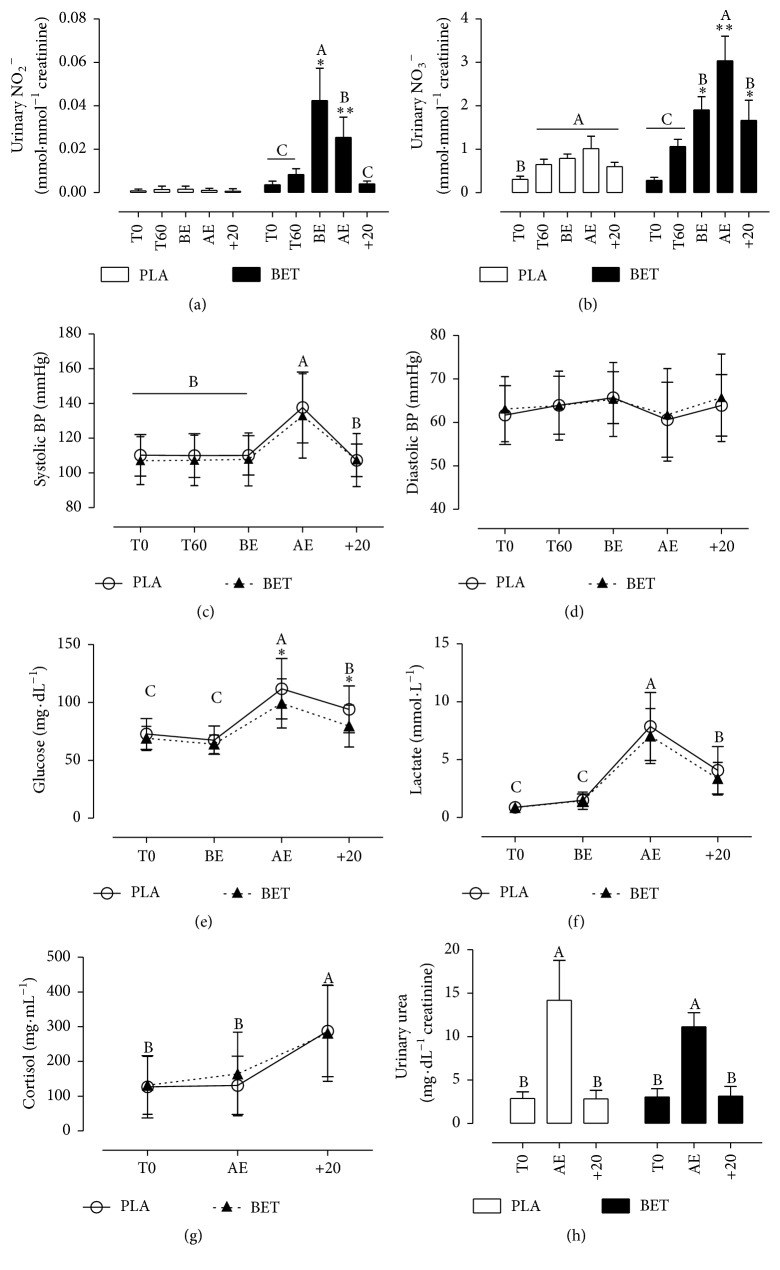
Urinary NO_2_^−^ (a) and NO_3_^−^ (b) concentrations, systolic (c) and diastolic (d) blood pressures, blood glucose (e), lactate (f), serum cortisol (g), and (h) urinary urea taken at the baseline (T0), 60 min after gel ingestion (T60), before exercise (BE), after exercise (AE), and 20 min after the end of the exercise (+20). The symbol ^*∗*^(*p* < 0.05) indicates significant difference compared to the PLA treatment. The symbol ^*∗∗*^(*p* < 0.001) indicates significant difference compared to the PLA treatment. Different letters denote statistical significance within the same treatment in different time intervals at *p* < 0.05. Values are presented as mean ± SD for urinary NO_2_^−^, NO_3_^−^, blood glucose, lactate, cortisol, urea, and mean ± SEM for hemodynamic parameters.

Urinary NO_2_^−^ concentrations were significantly higher after BET ingestion when compared to PLA treatment, before the exercise, BE (0.049 ± 0.018 versus 0.001 ± 0.000 mmol·mmol^−1^ creatinine), and even after exercise, AE (0.027 ± 0.011 versus 0.001 ± 0.000 mmol·mmol^−1^ creatinine) (Figure 2(a)). The NO_2_^−^ peak concentration occurred at 90 min, in BE (0.049 ± 0.018 mmol·mmol^−1^ creatinine), after supplementation and it was maintained until the end of the exercise. Urinary NO_3_^−^ concentration concentrations were significantly higher in the BET when compared to PLA treatment, BE (1.91 ± 1.43 versus 0.79 ± 0.47 mmol·mmol^−1^ creatinine), AE (3.03 ± 2.66 versus 1.01 ± 1.34 mmol·mmol^−1^ creatinine), and +20 (1.81 ± 2.12 versus 0.63 ± 0.56 mmol·mmol^−1^ creatinine) (Figure 2(b)).

### 3.3. Exercise Performance, BP, RQ, HR, and RPE

A single dose of BET did not promote significant differences in VO_2peak_ and time to fatigue when compared with PLA (Table 1). Systolic and diastolic BP did not differ significantly at any of the investigated time points (Figures 2(c) and 2(d)). Systolic BP was significantly higher after the BET and PLA treatments AE (133 ± 24.8 and 137 ± 21.2 mmHg) when compared to T0 (107 ± 13.7 and 110 ± 12.4 mmHg), T60 (108 ± 14.7 and 110 ± 13.0 mmHg), BE (107 ± 15.3 and 110 ± 11.6 mmHg), and +20 (107 ± 15.6 and 107 ± 9.8 mmHg). RQ, HR, and RPE measurements increased linearly with exercise intensity but did not differ significantly between BET and PLA treatments (Table 2).

### 3.4. Glucose and Lactate Concentration

Blood glucose concentrations were 11% higher (*p* < 0.05) AE (111.59 ± 24.25 mg·dL^−1^) and +20 (93.95 ± 19.32 mg·dL^−1^) for the PLA compared to BET supplementation AE (101.00 ± 21.79 and +20: 80.79 ± 18.11 mg·dL^−1^) (Figure 2(e)). Blood lactate did not differ between the treatments at any of the investigated time points (Figure 2(f)).

### 3.5. Cortisol Concentration

Serum cortisol concentrations before and after BET and PLA treatments did not differ between both treatments (*p* = 0.508) at any of the time points, although this hormone behaved as expected after exercise (Figure 2(g)). Times T0 (131.15 ± 83.38 and 127.22 ± 89.70 mg·mL^−1^) and AE (164.27 ± 120.02 and 131.44 ± 84.22 mg·mL^−1^) showed significantly lower contents than +20 (281.38 ± 138.54 and 287.90 ± 131.14 mg·mL^−1^) in both BET and PLA supplementations, respectively.

### 3.6. Urea Concentration

Urinary urea contents (*p* = 0.25) in BET and PLA treatments showed no significant differences in any time points. Urea levels were increased 3.6–4 fold after exercise (AE) but recovered to baseline levels (T0) after 20 min after both treatments (Figure 2(h)).

## 4. Discussion

Technological processes applied to foods can be advantageous to preserve, transport, store, and increase the nutritional value due to the concentration of compounds and nutrients. Foods in gel form are easier to drink and tolerable for endurance athletes before, during, and after exercise. The new formulated gels are composed of carbohydrates, fibers, saponins, and phenolic compounds (which contribute to their high antioxidant activity) and display adequate rheological properties, such as high viscosity, pleasant texture, and good consumer acceptance. Furthermore, the effect of BET has already been tested as an acute dietary NO_3_^−^ supplement with effects on NO synthesis and BP in five healthy subjects, where BET increased plasma NO_2_^−^ by 3 fold and decreased systolic and diastolic BP and HR after 60 min of ingestion [9]. Because of the advantages and beneficial effects related to food in gel form, the present study was developed with the objective of evaluating the effect of a single dose of nutritional BET gel rich in dietary NO_3_^−^ on the biochemical, metabolic changes, and performance on the practice of endurance sports.

Herein, a single dose of 100 g of BET gel was shown to increase final urinary NO metabolites, NO_3_^−^, and NO_2_^−^, following exercise. However, after 100 g of PLA gel ingestion, no changes were observed in urinary NO_3_^−^ and NO_2_^−^ concentrations. When it was ingested, dietary NO_3_^−^ is readily absorbed across the upper gastrointestinal tract, about 60% of NO_3_^−^ is excreted in the urine, and 25% is absorbed by salivary glands. In the oral cavity, the NO_3_^−^ is reduced to NO_2_^−^ by NO_3_^−^-reductase enzyme. After being swallowed, the NO_2_^−^ is decomposed nonenzymatically to NO. In aqueous solutions, NO undergoes rapid oxidation to NO_2_^−^ without the presence of oxyhemoproteins and thus NO_2_^−^ is the main product of NO decomposition [5, 11]. Therefore, it was expected the increase in urinary NO_3_^−^ concentration after BET and not in PLA gel ingestion because PLA gel had the dietary NO_3_^−^ removed. Also, that is because the urinary NO_2_^−^ concentration only increased after BET gel ingestion. For this reason, many studies have used urinary NO metabolites as an index of NO synthesis because NO_2_^−^ is metabolic end product derived from NO oxidation in plasma and subsequently excreted in urine [26].

The main finding of the present study is that despite increased NO synthesis after 100 g of BET gel ingestion no improvement in physical performance was observed, assessed by the reduction of VO_2peak_ and increase time to fatigue. This is in accordance with previous results, where the effects of a single and short-term (3–5 days) dose of NO_3_^−^ rich beetroot juice [27, 28] showed a very limited effect on VO_2_ and no improvements in physical performance. Arnold et al., [22] demonstrated that acute beetroot juice supplementation with 7 mmol of NO_3_^−^ did not promote any effect on VO_2peak_ and RPE after incremental exercise to exhaustion at 4000 m and a 10 km treadmill time-trial at 2500 m simulated altitude in well-trained competitive male runners. Also, Vanhatalo et al., [21] demonstrated that acute beetroot juice supplementation with ~5.2 mmol of NO_3_^−^ did not promote any effect on VO_2peak_ and GET in volunteers physically active. However, the same sodium NO_3_^−^ (NaNO_3_^−^) supplementation (0.1 mmol·kg^−1^·day^−1^) for 3 days reduced VO_2_ during submaximal cycling exercise in untrained men [29]. Beyond that, in a recent systematic review and meta-analysis [30], benefits on physical performance have been suggested as being more often and meaningful in healthy inactive individuals, rather than active individuals. This lack of positive results in physical performance observed herein and in the aforementioned studies conducted on volunteers well-trained for endurance can be explained by the physiological adaptations of endurance training that may play a role in increasing NO bioavailability by stimulating the expression and activity of NOS enzyme through the endogenous pathway (via L-arginine/NO) [31]. Due to the activation of the enzyme, the dependency of NO bioavailability derived from dietary NO_3_^−^ supplementation could be reduced. Comparing trained and untrained subjects, the trained ones are less prone to experience a low oxygen and muscle acidosis, which are favorable for the reduction of NO_3_^−^ to NO [32]. Therefore, any further stimulus for NO synthesis (like the NO_3_^−^-NO_2_^−^/NO pathway) can be reduced because of the high capacity of NO synthesis thought efficient endogenous pathways present in highly endurance-trained individuals.

Improvement in the physical performance of healthy active well-trained individuals was observed by the majority of studies, which offered long-term (6 or more days) beetroot supplementation with doses varying between 5.0 and 11.2 mmol of NO_3_^−^ [15, 19, 21, 29]. However, the precise mechanism by which NO_3_^−^ supplementation reduced VO_2_, implying improved muscle efficiency and enhanced exercise tolerance during physical activity, has not yet been elucidated. Given the multifarious roles of NO in physiology, it is not surprising that there are several possible explanations for the reported systemic effects of NO from NO_3_^−^ supplementation. Once synthesized NO can operate as a regulator of cellular O_2_ utilization increasing mitochondrial efficiency. The NO can then bind to O_2_ receptors, inhibiting the cytochrome c oxidase enzyme (complex terminal mitochondrial respiratory chain). Through inhibition of this enzyme, NO modulates intracellular and tissue O_2_ distribution [33]. In addition, NO may reduce the leakage of protons, attenuating the expression of uncoupled proteins, and thus increasing the oxidative phosphorylation efficiency [34]. Furthermore, decreases in O_2_ consumption can be attributed to the reduced ATP expenditure necessary to increase muscle contractile efficiency. During skeletal muscle contraction, the calcium pumping from the sarcoplasmic reticulum requires increased energy expenditure, and NO generated from NO_3_^−^ supplementation can increase the stoichiometric efficiency of the calcium-ATPase enzyme of the sarcoplasmic reticulum, increasing calcium transportation efficiency or preventing excess calcium release and reducing the energy consumption of its reuptake [34].

Aerobically trained individuals have specific physiological adaptations that may limit possible benefits from NO_3_^−^ supplementation. Also, one cannot exclude the possibility of “responsive” and “nonresponsive” individuals to NO_3_^−^ supplementation. Regarding systolic/diastolic BP and HR, no significant decrease was observed after the BET treatment. These results are in agreement with previous reports that found no significant changes in BP and HR in well-trained competitive male runners and recreationally fit volunteers after a single dose of beetroot juice and roasted beetroot containing 7 and 8 mmol of NO_3_^−^[18, 22]. However, studies in healthy but untrained subjects verified systolic BP reduction after a single NO_3_^−^ supplementation from beetroot [9, 21, 35]. It seems that, beyond the influence of acute versus chronic NO_3_^−^ supplementation (4–6 days), physical fitness may negate the effects of dietary NO_3_^−^ supplementation on BP, as quoted above.

The positive effect of NO_3_^−^ supplementation on lowering blood glucose was observed in the present study after exercising and during exercising recovery. Furthermore, as expected during high exercise intensity, blood lactate was increased in BET and PLA gels treatments. But, the lactate concentration did not differ significantly in BET and PLA gels at any time point, indicating that the aerobic exercise intensity was the same for both treatments. These findings corroborate previous studies [18] that also described lower plasma glucose in high intensity and intermittent exercise after beetroot juice supplementation (~8.2 mmol of NO_3_^−^) but no changes in lactate levels. NO plays a key role in the regulation of glucose uptake in contracting skeletal muscles by decreasing blood glucose during exercise, as GLUT4 translocation is enhanced [36].

As observed for blood lactate levels, urinary urea and serum cortisol concentrations did not differ significantly after BET and PLA gel ingestion. A late urea increment after 24 h could be expected, as observed after a 100 km race study [37]. No modifications in urea levels are expected after BET ingestion, since there was no protein intake during BET and PLA visits, eliminating interferences in the urea excretion in volunteers.

## 5. Conclusion

The new beetroot nutritional gel was successfully developed as a NO_3_^−^ supplement for athletes and showed to be advantageous for athletes as a replacement for sport drinks in order to avoid possible gastric discomfort due to the ingestion of large volume. Also, the gel is also easier to be transported and more appropriate to be consumed in sport events.

A single dose of this beetroot gel promoted an increased in the excretion of metabolites related to NO synthesis and promoted lower the plasma glucose immediately after exercise and during exercise recovery. However, the beetroot gel did not enhance the physical performance during aerobic submaximal exercise or cause changes in serum cortisol and blood lactate levels in recreational athletes. These results raise the question of the ergogenic potential of NO_3_^−^ to increase performance acutely in recreational athletes. Further acute NO_3_^−^ ingestion studies with more comprehensive data are needed to clarify if the NO_3_^−^ can actually improve physical performance in well-trained athletes.

## Figures and Tables

**Table 1:** Physical performance on the treadmill test: VO_2_ and time (s) after nutritional intervention with the BET and PLA gel supplementations.

Men and women	BET	PLA
	Moderate intensity	
Baseline VO_2_ (L·min^−1^)	1.43 ± 0.47	1.40 ± 0.44
Endpoint VO_2_ (L·min^−1^)	2.85 ± 0.65	2.89 ± 0.78
	Severe intensity	
Baseline VO_2_ (L·min^−1^)	2.88 ± 0.64	2.92 ± 0.81
Peak VO_2peak_ (L·min^−1^)	3.93 ± 0.84	4.00 ± 0.95
Time (s)	395.4 ± 179.60	390.90 ± 158.50

Values are presented as mean ± SD. BET, beetroot gel (9.92 ± 1.97 mmol·100 g^−1^); PLA, placebo gel (0.33 ± 0.15 mmol·100 g^−1^); VO_2_, oxygen volume.

**Table 2:** Physiological responses at moderate and severe intensities after the nutritional intervention with the BET and PLA gel supplementations.

Men and women	Group	Warm up	Moderate	Peak (severe)	*p*
RQ	PLA	0.77 ± 0.05	0.91 ± 0.05	0.97 ± 0.06	<0.0001
	BET	0.78 ± 0.06	0.91 ± 0.05	0.97 ± 0.06	<0.0001
HR (bpm)	PLA	98.46 ± 12.12	150.58 ± 11.81	179.58 ± 7.50	<0.0001
	BET	97.50 ± 10.92	150.85 ± 12.02	180.92 ± 10.88	<0.0001
RPE (Borg)	PLA	1.08 ± 0.28	4.12 ± 1.30	10.00 ± 0.00	<0.0001
	BET	1.08 ± 0.28	4.12 ± 1.24	9.88 ± 0.60	<0.0001

Values are presented as mean ± SD. BET, beetroot gel (9.92 ± 1.97 mmol·100 g^−1^); PLA, placebo gel (0.33 ± 0.15 mmol·100 g^−1^); RQ, respiratory quotient; HR, heart rate; RPE, rating of perceived exertion.
